# The Third Plague Pandemic in Europe

**DOI:** 10.1098/rspb.2018.2429

**Published:** 2019-04-17

**Authors:** Barbara Bramanti, Katharine R. Dean, Lars Walløe, Nils Chr. Stenseth

**Affiliations:** 1 Centre for Ecological and Evolutionary Synthesis (CEES), Department of Biosciences, University of Oslo, Oslo, Norway; 2 Division of Physiology, Institute of Basic Medical Sciences, University of Oslo, Oslo, Norway; 3 Department of Biomedical and Specialty Surgical Sciences, University of Ferrara, Ferrara, Italy

**Keywords:** *Yersinia pestis*, hygiene, human ectoparasites, *Rattus rattus*

## Abstract

Plague has a long history on the European continent, with evidence of the disease dating back to the Stone Age. Plague epidemics in Europe during the First and Second Pandemics, including the Black Death, are infamous for their widespread mortality and lasting social and economic impact. Yet, Europe still experienced plague outbreaks during the Third Pandemic, which began in China and spread globally at the end of the nineteenth century. The digitization of international records of notifiable diseases, including plague, has enabled us to retrace the introductions of the disease to Europe from the earliest reported cases in 1899, to its disappearance in the 1940s. Using supplemental literature, we summarize the potential sources of plague in Europe and the transmission of the disease, including the role of rats. Finally, we discuss the international efforts aimed at prevention and intervention measures, namely improved hygiene and sanitation, that ultimately led to the disappearance of plague in Europe.

## 1. Introduction

Ancient DNA studies have identified *Yersinia pestis,* the aetiological agent of the Third Pandemic, as the cause of the previous plague pandemics: the First Pandemic (sixth to eighth centuries) [1–3] and the Second Pandemic (fourteen to nineteenth centuries) [4–8]. During all three pandemics, distinct strains of *Y. pestis* were introduced to Europe causing epidemics of plague, including the infamous Black Death (1346–1353); the strains from the first two pandemics are now extinct. Recently, researchers have identified the earliest known strains of *Y. pestis* in Europe dating as far back as the Stone Age [9–11].

While plague clearly has a long history in Europe, there are no known reservoirs for the disease today [12], which has generated debate surrounding how the ecology and epidemiology of plague has changed over time [13,14]. Here, we investigate plague during the Third Pandemic in Europe, as it differs from other parts of the world, in order to characterize the unique epidemiology of the disease during this time period.

The Third Plague Pandemic originated in the Yunnan region of southwest China, where plague caused multiple outbreaks since 1772 [15–17]. In 1894, plague reached Canton and then spread to Hong Kong, where Alexandre Yersin identified the bacterium.

It was then carried by ships to Japan, Singapore, Taiwan and the Indian subcontinent [18,19]. Over the next few years, plague spread to many cities around the world: Bombay, Singapore, Alexandria, Buenos Aires, Rio de Janeiro, Honolulu, San Francisco and Sidney, among others [20]. The earliest known European cases occurred in September and October 1896, when two sailors from Bombay died of plague on ships docked in London on the Thames [21].

Case records and outbreak reports for the Third Pandemic are numerous and have improved our understanding of the historical epidemiology and distribution of plague. These reports have been compiled and summarized for several regions: North America [22,23], South America [23,24], Africa [23,25] and Asia [23]. However, a similar account for Europe is missing, making it difficult to compare local and global transmission patterns. Europe is also the only region for which we have extended records and accounts on the previous plague pandemics, in particular those of the Second Pandemic. Thus, having documented outbreaks of the Third Pandemic can enable comparisons with historical ones, especially considering that the Third Pandemic in Europe was restricted to the pre-antibiotic era.

Here, we compile the reported plague cases for Europe during the Third Pandemic from digitized records of notifiable diseases, previous studies and grey literature. We describe important cases and outbreaks that took place during the Third Pandemic and the international efforts enacted to prevent the importation and spread of the disease. We also investigate the role of rats and other sources of plague, which contributed to decades of small outbreaks. Finally, we discuss the eventual disappearance of plague in Europe owing to increased hygiene and a lack of a long-term rodent reservoir.

## 2. Methods

We systematically collected data for plague cases in Europe from the *Public Health Reports* (formerly *Bulletins of the Public Health* and *Weekly Abstract of Sanitary Reports*) between 1879 and 1950 accessed through PubMed Central (https://www.ncbi.nlm.nih.gov/pmc/). In the original reports, cases in the period before September 1927 were recorded mainly as outbreaks with start–end dates and those after September 1927 were recorded as weekly or monthly incidence. For some of the early outbreaks, such as those in Porto (1899) and Glasgow (1900), the cases are more temporally resolved. We present these raw data in the electronic supplementary material, table S1 (1899–1927 in blue, 1927–1947 in green), with the highest resolution available from the reports. For overlapping reports, we used the most recent in time, corresponding to the highest number of cases and deaths. Our study area was continental Europe, excluding Russia, but including the Mediterranean islands. We excluded Russia because their reporting of cases internationally has been sparse and irregular. We converted city and country data to latitudes and longitudes for mapping using GeoPy (https://geopy.readthedocs.io/en/stable/).

We used narrative and scientific reports in four languages (English, French, Italian and German) to supplement the case data. These reports are translated and summarized in the electronic supplementary material. The reports consisted of primary accounts, secondary accounts and scientific reports, which are mainly found in grey literature.

## 3. Results

**Figure 1.**
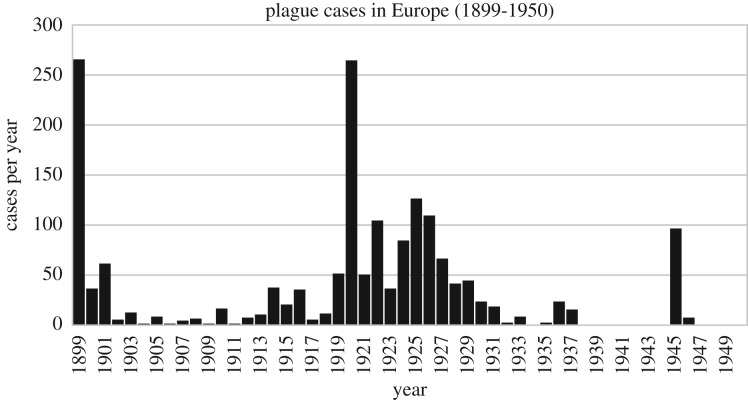
Reported suspected plague cases per year in Europe (1899–1950) from the *Public Health Reports*. See also the electronic supplementary material, table S1.

**Figure 2.**
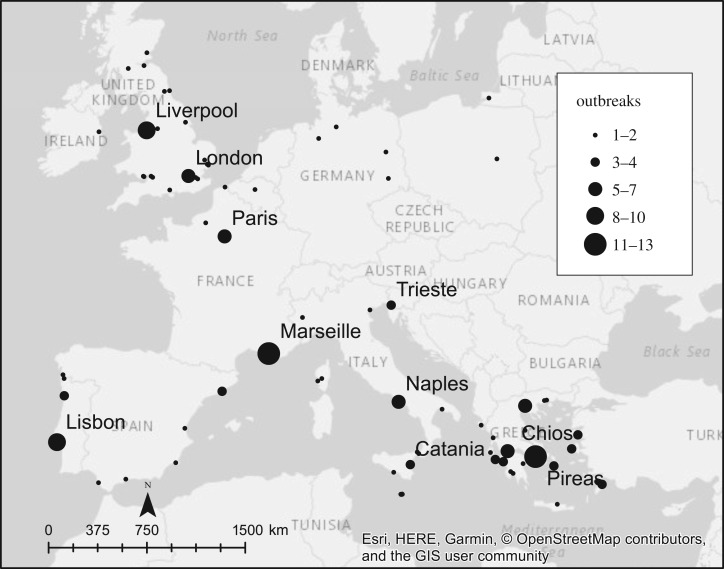
Map of reported plague cases in Europe (1899–1947) from the *Public Health Reports* and electronic supplementary material, including the number of outbreaks in each location (see also the electronic supplementary material, table S1).

There were 1692 cases and 457 deaths from plague reported in Europe between 1899 and 1947 (figure 1; electronic supplementary material, table S1), with the largest number of cases in the years 1899 and 1920. Cases were geographically widespread, although they were primarily found in coastal or inland port cities (figure 2). Plague was reported in 11 countries, and many cities, including Lisbon, Marseille, Paris and Pireas, experienced multiple outbreaks (table 1). Plague was notably absent in some parts of Europe. For instance, the Nordic countries, which reported infectious diseases such as polio and cholera, did not report any plague case during the Third Pandemic.

**Table 1.** Locations and years of reported plague outbreaks in Europe (1899–1950) from the *Public Health Reports* and electronic supplementary material. (Only locations with multiple plague outbreaks are shown (see also the electronic supplementary material, table S1). International Organization for Standardization (ISO) Country code in parentheses.)

location	years
Athens (EL)	1913, 1915, 1919, 1920, 1925, 1926, 1927, 1928
Avonmouth (UK)	1919, 1931
Barcelona (ES)	1902, 1919, 1922, 1931
Catania (IT)	1914, 1920, 1921, 1922
Chios (EL)	1893, 1914, 1916, 1920
Dublin (IE)	1920, 1921
Dunkirk (UK)	1902, 1922
Glasgow (UK)	1900, 1901, 1907, 1908
Hull (UK)	1901, 1916
Lisbon (PT)	1899, 1910, 1914, 1920, 1921, 1922, 1923, 1924, 1926, 1928
Liverpool (UK)	1901, 1905, 1908, 1912, 1914, 1916, 1919, 1920, 1926
London (UK)	1900, 1905, 1910, 1917, 1918, 1919, 1920
Marseille (FR)	1902, 1903, 1907, 1919, 1920, 1924, 1925, 1926, 1930, 1932, 1933, 1935, 1936
Mytilene (EL)	1927, 1928, 1929, 1930
Naples (IT)	1901, 1921, 1922, 1924, 1929
Paris (FR)	1920, 1921, 1922, 1923, 1924, 1926, 1929
Patras (EL)	1922, 1924, 1925, 1926, 1927
Pireas (EL)	1913, 1914, 1915, 1916, 1919, 1920, 1921, 1922, 1925, 1926, 1927, 1929, 1930
Porto (PT)	1899, 1900, 1923
Pyrgos (EL)	1925, 1929, 1930
Rhodes (EL)	1910, 1921, 1925
Saint-Ouen (FR)	1926, 1930
Syros (EL)	1914, 1916, 1923
Taranto (IT)	1927, 1945
Thessaloniki (EL)	1914, 1915, 1919, 1920, 1924, 1925
Trieste (IT)	1906, 1908, 1912, 1913
Zakynthos (EL)	1915, 1920, 1926

From a comparison with the grey literature summarized in the electronic supplementary material, it is evident that not all cases have been reported in the *Public Health Reports*. For instance, the last outbreak in Taranto in 1945, with 30 cases and 15 deaths, was hidden owing to military reasons, and possibly, other cases were not reported in times of war. We see that cases were mainly notified in large cities and ports, which had more traffic from trade but also may have had more resources and established practices for detecting infectious diseases. Some regions, such as the Nordics and Eastern Europe, did not report any case of plague. While plague may be truly absent in these areas, we cannot exclude the possibility that plague was undectected or unnotified. Nevertheless, over-reporting may have occurred if cases were misdiagnosed as plague. While early bacteriological methods were used to identify plague in some instances, to our knowledge, most of the cases in the electronic supplementary material, table S1 were not confirmed. Official reports and accounts of individual outbreaks such as those in Oporto, Glasgow and Taranto (summarized in the electronic supplementary material) offer more detailed information about case numbers, symptoms, transmission and mortality, which may differ from the information in the *Public Health Reports* and electronic supplementary material, table S1.

## 4. Discussion

During the later part of the nineteenth century, diseases such as cholera and later plague were spreading throughout the world, partly owing to the advent of steamships [26]. This necessitated the development of adequate measures to prevent the introduction and spread of infectious diseases to Europe. The European sanitary authorities responded by meeting often to discuss preventative measures against plague and other diseases. International conferences were held in Venice in 1892, in Dresden in 1893 and in Paris in 1894 [21].

Two events emphasized the re-emerging threat of plague to Europe in the late 1800s. The first was an outbreak of pneumonic plague in Vetlianka, along the Volga River, in Russia [21]. Three commissions were sent to nearby Astrakhan by European governments (French, British and joint Austrian–German) to study the outbreak which resulted in more than 400 cases [21,27–31]. The second event was the discovery of two sailors from Bombay who died of plague on a ship in London in 1896 [21,32]. These events prompted European officials to convene an international sanitary conference in February 1897 in Venice to specifically discuss the spread of plague [21]. Another key international plague conference was held in Shenyang (old name, Mukden) in April 1911, with epidemiologists and scientists from 11 countries (China, Japan, USA, Great Britain, France, Germany, Italy, Austria–Hungary, The Netherlands, Russia and Mexico) [33]. The conference was chaired by Dr Wu Lien Teh, who had stopped the great epidemic of pneumonic plague in Manchuria and Mongolia (about 60 000 victims) by 1910 [33].

Following the international conferences, regular reporting of infectious diseases in Europe began in the 1890s [34]. For plague, detailed records of cases and deaths appear in the *Public Health Reports* beginning in 1899 (electronic supplementary material, table S1). These reports show that plague was continually introduced to European ports throughout the Third Pandemic by ships arriving from abroad, often from the former European colonies such as Bombay, Buenos Aires and Alexandria (electronic supplementary material, table S1). Ships arriving in European ports, such as those in the UK, were checked for early signs of plague at arrival and filled out a ‘Declaration of Health’ [35]. These early signs of plague included suspicion of human or rat cases onboard, as well as unexplained rat mortality [35], which was also noted in many of the case reports (electronic supplementary material, table S1). It appears that plague was also transported by other means, as there are several accounts relating to specific cargo, such as clothing, rags, grain and other merchandize probably containing infected rats or fleas [20,21,32,36–44].

It is clear from the prevention measures enacted that the authorities were aware of the role of maritime trade in the spread of plague (e.g. [21,35]). For instance, in Venice in 1897, they organized quarantines, controlled maritime traffic from infected areas without stopping trade, and regulated the hygienic condition of ships, travellers, crew and goods entering Europe. It was noted by Proust that, ‘As in the previous meeting about cholera, it was decided that the treatment applicable to ships must be regulated by their sanitary condition at the arrival and not by the state of the port of provenance which gives only indications, which may be valuable indications but which are only indications. This is the new principle underlying modern international prophylaxis' [21, p. vi]. The recommendations of the conference to governments resulted in a complex system of regulations that controlled carriers coming by land and sea from infected regions [21]. Despite the regulations in place, Europe experienced several outbreaks of plague during the Third Pandemic, but the vast majority of these outbreaks were small (electronic supplementary material, table S1).

### (a) Role of rats and other sources of plague

**Figure 3.**
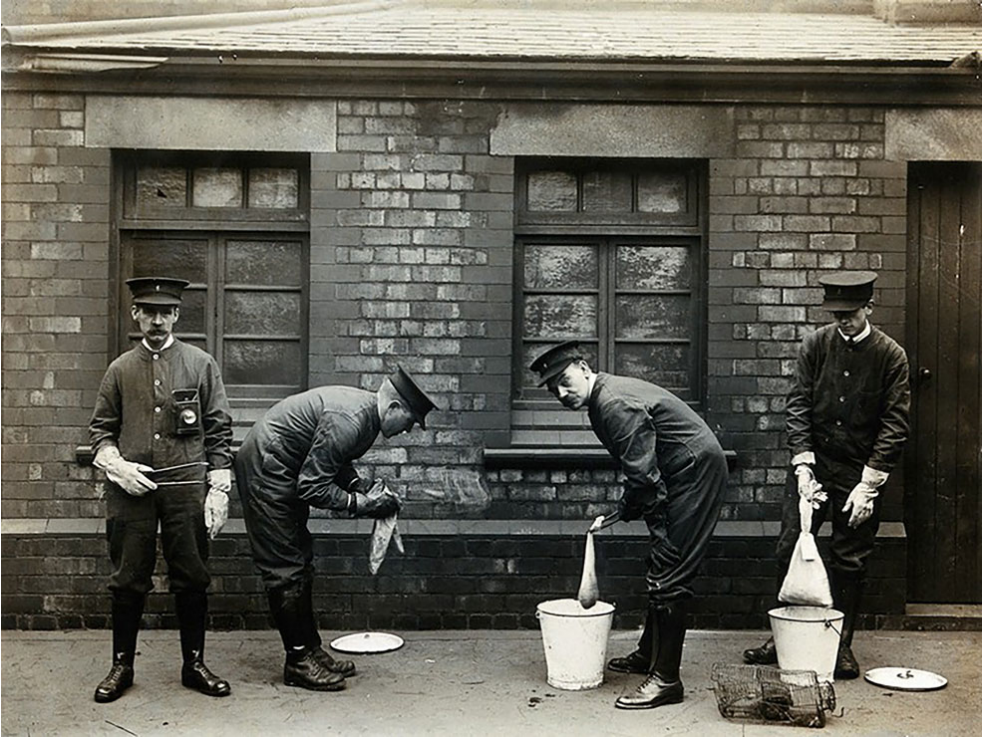
‘Liverpool Port Sanitary Authority rat-catchers dipping rats in buckets of petrol to kill fleas for plague control. Liverpool, England. Photograph, 1900/1920’. Image courtesy of Wellcome Collection. Credit: Wellcome Collection. CC BY 4.0.

At the beginning of the Third Pandemic, physicians and scientists used new methods to increase their knowledge of plague, including microbiological and experimental techniques [45]. From the late 1800s, Lowry [46], Rocher [47] and Yersin [48], among others, observed a connection between human and rat plague mortality during epidemics in India and China, suggesting that black rats were involved in transmission. This observation was later confirmed by Simond, who demonstrated in 1897 that rat-fleas were vectors for the disease [49,50]. The prevailing view among researchers in the Indo-Pacific region, including Thompson [51] who observed plague outbreaks in Sydney, Hunter who reported on plague in Hong Kong [52] and those of the Indian Plague Commission [53], was that black rats played an important role in the spread of plague, both as hosts in the chain of transmission and as carriers of the disease on ships [54]. When plague was introduced to Europe during the Third Pandemic, rats were heavily scrutinized by European health authorities (figure 3) when plague cases were discovered (e.g. [36,37,55], see also the electronic supplementary material).

There were two species of commensal rats present in Europe during the Third Pandemic, the black rat (*Rattus rattus*), also called the ship rat or the roof rat, and the brown rat (*Rattus norvegicus*), also called the sewage rat. The black rat has a history in Europe dating back to medieval times, but it has never been present in large numbers because the climate in Europe is too cold for it to be able to live and reproduce outside heated buildings [56]. The brown rat came to Europe from Russia during the early part of the eighteenth century and was abundant in all European cities around 1900 [57,58]. The two species are similar in appearance, but they have very different behaviour, as first described in a German zoological journal in 1952 by Eibl-Eibesfeldt [59] and later in great detail by Telle [60]. The American zoologist Davis [56] described similar differences in articles from the mid-1950s. The British zoologist Twigg later describes these differences in his book on ‘The Black Death’ [61]. These sources state that the black rat is an efficient climber, which makes nests in the walls and roofs of buildings, while the brown rat may live outdoors in the European climate, is an efficient swimmer, and makes nests in borrows in the soil, in cellars or in sewage pipes [57,59]. The two species of rats carry the same species of fleas. Owing to their different behaviour, black rats are living closer to humans than brown rats. During the Third Pandemic, plague was transported around the world by black rats on ships. At this time, black rats were not generally found in Europe, except in warehouses in ports and in a few towns [62].

From the first reports of plague, European sanitary authorities actively searched for dead rats in cities [39,63–66], urban districts [36,44], isles [67–70] and on ships [19], and they used early bacteriological methods to test for the plague bacterium in the local black and brown rat populations (e.g. [36,39,43,63–68,70]). For instance, when plague broke out in Glasgow in 1900 (see the electronic supplementary material), the Medical Officer of Health caught and examined 326 rats, but found no evidence of plague in the rat population [39,63]. They wrote after the outbreak that ‘inquiry failed to discover any evidence that rat mortality prevailed to an unusual extent’ [63, p. 26]. However, in the years following the outbreak, they found some evidence of plague in the rat population: in 1901 (122 of 1641), in 1902 (30 of 6492) and in 1907 (1 of 140) [55].

Rats were also examined during and after outbreaks in East Suffolk [36,44], Malta [67–69], Italy [66], Corsica [70], Spain [65] and France [64] (see the electronic supplementary material). After a small outbreak of plague in Taranto, Italy, in 1945, there was a large-scale anti-rodent campaign, which killed around 5000 rats [42]. Of these, 60% were *R. norvegicus* and 40% were *R. rattus* in the docks, while all of the rats in the city were black rats. In 1945, they found only two rats tested positive [66] and, in 1946, none were infected [43]. There was a similar outbreak in Ajaccio, Corsica, on 12 May 1945, with 13 cases of plague reported over 10 weeks [71]. It was rumoured that dead rats were observed before the outbreak, but none were examined. Following the outbreak, the authorities trapped 148 rats, 14 were *R. rattus* and the rest were *R. norvegicus*, but they found no evidence of plague [70]. Rat monitoring was also carried out in Marseille, France, where 132 cases of plague were reported from 1919 to 1929 [64]. The largest rat epizootic found in Marseille occurred in the poor downtown areas in 1930, where 42 infected rats were discovered out of the 7275 that were examined [64].

Perhaps the most extensive rat surveys carried out during the Third Pandemic in Europe were in and around East Suffolk, Britain, where cases appeared regularly from 1906 to 1918 [36,44]. The pattern of recurrent cases in East Suffolk led researchers John and Dorothy Black to assume that plague was endemic in this region [36]. Surveys for plague were carried out over an area of more than 2000 km^2^ [36,44]. However, only 60 plague-infected rats were found out of more than 266 000 rats that were caught during the 3 year survey [36,44]. In addition to rats, the authorities found some ferrets, cats and rabbits that died of plague [44]. The local authorities concluded that the infected rats were most probably brought by grain ships which unloaded their cargo in the area to lighten their draught before continuing onwards [36,44].

Other documented sources of plague in Europe were from direct human transmission of pneumonic plague (e.g. [36,44,63]) and the transportation of infected vectors (e.g. [36,63]) (electronic supplementary material, table S1). Pneumonic plague occurs when plague infects the lungs, either primarily by the spread of infectious droplets or secondarily as a complication of bubonic plague. Cases of pneumonic plague were reported during many of the outbreaks in Europe (electronic supplementary material, table S1) and often spread within households and among close contacts [36,44]. For example, in East Suffolk, a 9-year-old girl became ill with pneumonic plague and died in a cottage 5 miles from Ipswich on 13 September 1910 [36,44]. Her mother also contracted the disease and died 3 days after her daughter's death, followed by her stepfather and a neighbour who nursed her mother. To prevent further spread, the funeral services of the victims were held in open air and the contacts of the deceased were isolated [36,44].

There are also accounts of bubonic plague transmission without a clear association with rats, probably from infected vectors (e.g. [39,63,72]). Many different flea species can carry and transmit plague, such as those commonly found on rats (*Xenopsylla cheopis*), cats (*Ctenocephalides felis*) and humans (*Pulex irritans*) [21]. Ectoparasites were so abundant in Europe that the Third International Congress on School Hygiene held in Paris in 1910 advised to fight against them, because one out of every three children was infested [73]. As it is still the case for today, vermin infestations back then were associated with poverty and unhygienic living conditions (e.g. [36,63,64,67,74]), often in the poorest quarters of cities, where the majority of cases were found during outbreaks such as Oporto (1899), Glasgow (1900) and Marseille (1900–1921). Scheube wrote that, ‘The development and spread of plague is influenced in a great measure by the *unfavorable hygienic conditions, essentially connected with social misery*’ [75, p. 12]. In some cases, it appears that infected vectors transmitted the disease between people in close contact. For example, during the plague in Glasgow in 1901, a woman who had fallen ill with the plague was visited by two friends from Liverpool [38] (see the electronic supplementary material). Weeks later in Liverpool, a chain of deaths from plague began among the relatives and neighbours who handled the clothes worn by the two girls in Glasgow [38]. Indeed, infected ectoparasites in clothing, rags, grain sacks and other textiles could explain the appearances of plague even in the absence of infected rats (e.g. [21,38,63]).

Overall, the connection between urban rodents and human plague in Europe during the Third Pandemic is less clear than for outbreaks in India and China [21,46,48–50,54,75–78]. However, it was often proposed that other sources of plague, such as infected human-specific or human-biting parasites, like fleas and lice, were important for transmission in Europe during the Third Pandemic [21,36,63,64,74]. The low numbers of plague-infected rats found during European outbreaks suggest that they played a relatively minor role in plague transmission. However, some researchers have argued that the authorities were unlikely to find plague-infected rats because they would go into hiding [51], thus differing in their behaviour from the rats in Hong Kong during the outbreak of 1894, which were described as dead ‘in abundance on the streets and in the houses' [48, p. 311]. The low number of human plague cases in Europe during the Third Pandemic could be explained by a low number of infected rats, but it could also be a reflection of effective public health intervention measures that reduced the contact between humans and infected vectors, such as isolation of patients and contacts, prohibition of gatherings and improved hygiene (e.g. [21,63,72]).

### (b) Disappearance of plague

Plague is not a disease that is found in Europe today, and we found no mention of plague outbreaks after 1950. The disappearance of plague in Europe during the Third Pandemic can be attributed to two main factors, improved hygiene and the lack of a present-day sylvatic reservoir for the disease.

At the end of the nineteenth century, the newly established discipline of microbiology found causative relationships between germs and diseases. In 1897, Proust observed that, ‘It is no matter of doubt that the plague cannot produce nowadays the disasters of the Black Death in the 14th c. Fortunately, the general hygienic conditions have much changed’ [21, pp. 1–2]. Indeed, during the nineteenth century, the spread of several diseases like tuberculosis, smallpox, cholera and yellow fever prompted extensive campaigns in European cities to improve hygienic conditions [79]. In many places in Europe, this work included the destruction of slums, improvement of sewage systems and the widespread development of safe water supply systems [79].

Contemporary scholars regarded cleaning and disinfecting as an essential part of plague control measurements [21,67,80]. Proust described in Bombay that in places where it was possible to clean dwellings, houses and streets, plague outbreaks could be contained or avoided [21]. Indeed, from the 1950s, the introduction of baths in the majority of European dwellings, and the use of vacuum cleaners and washing machines, strongly enhanced personal hygiene and that of the domestic environment (e.g. [81]). In addition, from the middle of the twentieth century, the number of pests and parasites was reduced by the introduction of insecticides like dichlorodiphenyltrichloroethane (DDT), which was used heavily in many places like Malta from 1946 onwards [69]. In Taranto in 1945, the allied forces, contributed noticeably to the fight against the epidemic by spraying large quantities of DDT against ‘fleas, but also bugs, lice and ticks' [66, p. 167].

Although the existence of a rodent reservoir for plague in the past is heavily debated [8,14,17,56,82–84], there is no evidence that plague is endemic to Europe today or was at any time during the Third Pandemic. Introductions of plague during the Third Pandemic led to the formation of plague reservoirs in the USA [22,23], South America (Peru, Bolivia and Brazil) [23,24] and Africa (Democratic Republic of the Congo, Tanzania, Uganda and Madagascar) [23,25], where ecological conditions have favoured the persistence of the bacteria in sylvatic rodent communities. Today, the spillover of plague from these reservoirs leads to the thousands of cases of plague reported every decade [85]. However, not all introductions of plague led to the formation of reservoirs, typically found in arid and semi-arid highlands [17], which are not present in Europe. The lack of a rodent reservoir in Europe is the fundamental reason why plague is no longer a public health threat today on the continent. The unfavourable environmental conditions in western Europe make it very unlikely that there has been a wild plague reservoir there. Even in Malta, where the environment is much more favourable to rodent reproduction [67], Barnett observed that ‘plague outbreaks always come to an end even if nothing is done to kill rats or their fleas’ [67, p. 17]. It is possible that future ancient DNA studies will demonstrate that all of the different lineages of *Y. pestis* involved in historic outbreaks went extinct after their introduction into Europe (see also Namouchi *et al*. [4]).

## 5. Conclusion

Although plague is no longer a public health issue in Europe today, the threat of the disease remains close in both space and time. Plague was in Europe until the middle of the last century, just two generations ago. The disease has recurred in Algeria [86] and Lybia [87] less than a decade ago, in places that are less than 300 miles from European boarders. Moreover, plague is currently present in 11 countries around the world [85]; at a time of globalization, characterized by the increased mobility of people and goods, diseases can easily spread from endemic or enzootic regions (i.e. foci and reservoirs) to the rest of the world in a short time [88]. A recent paper [89], which analysed plague cases reported since the end of the last century, has proposed classifying plague as a re-emerging disease. Indeed, in the last years, the frequency of plague outbreaks in developing countries in Africa should not be overlooked; industrialized countries must react promptly to plague outbreaks as well as other epidemic diseases, in order to inform the population and help fight against them.

## Supplementary Material

Dataset

## Supplementary Material

Electronic Supplementary Material

## Data accessibility

The datasets supporting this article have been uploaded as part of the supplementary material (electronic supplementary material, table S1).
