# Supplementary material for: The Third Plague Pandemic in Europe
Source: Proc Biol Sci. 2019 Apr 17;286(1901):20182429. doi: 10.1098/rspb.2018.2429 (PMC6501942; doi:10.1098/rspb.2018.2429)
Supplement: Electronic Supplementary Material [file rspb20182429supp2.pdf]

Electronic Supplementary Material

Proceedings of the Royal Society B
DOI: 10.1098/rspb.2018.2429

The Third Plague Pandemic in Europe

Barbara Bramanti[1,2*#,] Katharine R. Dean[1*,] Lars Walløe[3], Nils Chr. Stenseth[1#]

*The two authors have equally contributed.

[1]Centre for Ecological and Evolutionary Synthesis (CEES), Department of Biosciences, University of Oslo, Oslo, Norway

[2]Department of Biomedical and Specialty Surgical Sciences, University of Ferrara, Ferrara, Italy

[3]Division of Physiology, Institute of Basic Medical Sciences, University of Oslo, Oslo, Norway

[#]Corresponding authors: barbara.bramanti@ibv.uio.no and n.c.stenseth@ibv.uio.no

ACCOUNTS OF PLAGUE IN EUROPE DURING THE THIRD PANDEMIC

London 1896. This outbreak is the first known importation of plague into Europe during the Third Pandemic. Proust[1] reported that plague was discovered on two vessels docked on the River Thames. The disease spread, and on the 26th or 27th of September, a storekeeper's helper fell ill and later died on the 3rd of October. A second helper also became ill on the 26th of September and died on the following day. Another infected ship arrived on the Thames on the 7th of September. After taking on crew a few months earlier in Bombay, the ship left Calcutta and stopped at Colombo, Aden, and several other ports before arriving on the Thames. On the 16th of September, an Indian crewmember fell ill and his condition worsened for two or three days until he died on the 19th at hospital.

Conference of Venice on February 16th 1897. Details of the public health measures proposed at the Health Conference of Venice on February 16th 1897 are given in a renowned and comprehensive report by Proust[1]. Many of these measures were targeted specifically at preventing the spread of plague to Europe. For instance, regions outside of Europe that were under the threat of plague, i.e., plague foci, were to be monitored. In particular, this included areas of Iraq and Iran. Ports were also kept under surveillance, as well as border crossings, where plague could be imported from Russia, India, Afghanistan, and Pakistan (Belochistan). For ports, the conference suggested strong measures, including compulsory daily medical examination of all people on board ships, by a doctor delegated by a public authority. In addition, the measures suggested rigorous disinfection of any objects that were suspected of harboring plague. For overland travel, measures were to be taken during transport through provinces with plague and were meant to comply with the rules accepted in Venice in 1892, in Dresden in 1893, in Paris in 1894, and in Venice in 1897. Modern sanitary practices were to replace quarantine for travelers, including placing ovens and other disinfection stations

along well-traveled routes and railways. Goods were to be disinfected according to the principles adopted by the Conference of Venice of 1897. The conference agreed to rigorous measures of border closure in exceptional cases, with the option for governments to close their borders to passengers and cargo.

Vienna 1898. Three people died of plague after cultivating bacteria in the laboratory, brought from Bombay and confirmed to be *Y. pestis*[2].

Oporto 1899. Two weeks after plague was officially declared in Alexandria, Egypt, plague arrived in Oporto and gave rise to the first large European outbreak during the Third Pandemic[3]. Plague had vanished from Oporto for two centuries. The disease was reintroduced to the city by goods[2] or rats, imported by a ship from Bombay[4]. The first five patients were Galician laborers, who unloaded a shipment of wheat of unspecified origin on the 5th of June[2,3]. Five women who had been hired to sew and mend the grain sacks also died of plague[3]. It was not until the 20th of July that plague was confirmed by bacteriological examination for the first time. This was reconfirmed on the 8th of August, but the government did not declare a public health emergency until the 23rd of August[3]. As a result, plague spread until the beginning of February 1900, and the official statistics cited a total of 322 cases and 115 deaths. The number of deaths was likely higher, given that the total mortality in Oporto rose from an average of 4,650 deaths to 5,520 in 1899[3]. The outbreak in Oporto caused considerable alarm, as evidenced by the many medical commissions sent from around the world to assist with the outbreak[5]. Those working to stop the outbreak were among the victims, including the famous physician Luís da Câmara Pestana[6], a Professor of Anatomo-pathology and Legal Medicine in Lisbon, who had been in Oporto studying the nature and symptoms of plague.

The majority of the victims were from poor waterfront neighborhoods, including: Sao Nicolau, A Sé, Sao Ildefonso, Victoria and Miragaya, and surrounding villages. In the more prosperous commercial and residential quarters of the city, fewer cases were reported and, among those, nearly all were domestic servants, day laborers, and shop clerks[3]. It was written that, “Porto offered horrendous living conditions for its working poor”[3]. A. Shadwell, an official British medical observer, described the living conditions in Oporto in 1899:

> The city center was overcrowded with recent arrivals who had doubled its population in the last three decades of the nineteenth century: The city had just installed electric lighting and begun building a tram network, but in 1899 a modern sewage system did not yet exist. In its poorest districts closest to the port, the visitor could observe oxcarts, sedan chairs, and wagons drawn by as many as ten mules. Here, residents of the squalid tenements were subjected to some of the highest rates of mortality per thousand recorded anywhere in Europe… In addition to the standard measures used elsewhere, two antiquated and controversial procedures were applied by the Federal Board of Health in Lisbon: the erection of a military cordon sanitaire around Oporto and the imposition of official censorship on all information concerning the plague emergency[3].

The measures enacted against plague were in violation of the Venice protocols of 1897. Citizens reacted against them and some soldiers disobeyed orders and broke the blockade. In a letter dated December 8th, 1899, Surgeon Fairfax Irwin wrote:

> The sanitary cordon around the city is inefficient owing to the poverty of the soldiers and their inability to withstand the bribes offered them by the country people wishing to pass through. It is doubtful if this sanitary cordon now exists, as the people of Oporto were on the verge of revolution on account of the restrictions to trade and travel and the probability that a change of ministry would result in the withdrawal of the cordon[7].

Unsurprisingly, wealthy citizens, numbering as many as 20,000-30,000 people, fled the city. All sanitary restrictions put in place during the months that plague ravaged the city were eventually suspended by decree on the 6th of February, 1900[6].
As the first large outbreak during the Third Pandemic, "Porto was the first city where physicians used extensive serum and vaccine therapy in response to an outbreak of plague"[3]. One hundred forty-two patients received the Pasteur serum to prevent plague and, of them, 21 died. However, it was not known if the vaccine offered protection against the disease because the experiment was not conducted with a control group[3].

Glasgow 1900. In autumn 1900, plague reappeared in Glasgow after two and a half centuries. The outbreak consisted of 36 cases, of which 16 were fatal[8]. The earliest known cases were described in a report about the outbreak:

> A child and its grandmother (Mrs. B.), living in the same house at 71 Rose Street, South-Side, Glasgow, sickened suddenly on the evening of 3rd August — the child dying on the 7th and the grandmother on the 9th — the cause of death of the child being certified as "zymotic enteritis,'' and of the grandmother "acute gastro-enteritis". […] In both cases a wake was held, and the grandmother was buried on the 11th. Although the husband of this latter patient sickened on the 12th, he was only admitted to hospital on Monday, 27th August, certified "enteric fever," when he was recognized to be suffering from plague[8].

Care was used by the sanitary commission to ascertain the origin of plague, as described in the report:

> The house occupied by the B. family was a single apartment on the ground floor. It is distant at least a quarter of a mile from the river — considerably further from the docks. The father, although a dock laborer, was employed exclusively in vessels engaged in the coasting trade, and no evidence of other association with shipping could be found. The mother was a fish hawker, and took special charge of her grandchild. This is important, because the grandmother took the child with her wherever she went, and they sickened simultaneously. It suggests that they found their infection beyond the limits of their dwelling. […] The only other inmate of this house was a daughter — mother of the baby referred to — and employed, until the date of her mother's sickening, in a rag store. She was not affected. […] Concurrently with the later developments in this household, the following illnesses were appearing in the members of a family (M.), 57 Thistle Street, some of whom had either attended Mrs. B.'s wake, or were present during the illnesses in her house[8].

The sanitary authorities constructed a chain of transmission among the contacts of the initial cases. However, not all cases could be connected to previous ones. In general, the disease spread in the poor quarters of the city, where there was overcrowding in dwellings with poor light and ventilation. However, in one case a woman was infected without any direct contact with these areas. She was the wife of a clothes collector, who cleaned the personal belongings of the plague victims. It was noted in the report that, "the houses of the majority of the cases were hotbeds of vermin, and the clothes collector, like all those who had to deal with the infected houses, frequently complained of the annoyance these insects caused him"[8]. The clothes collector had received a dose of Yersin's serum and did not develop plague, but it was thought that he transported the parasites home with the clothes[8].

The mechanism for the transmission of plague was not clear to the sanitary authorities. They regarded wakes with particular suspicion, as many of the cases were connected through contacts made during wakes. They wrote that:

> Waking, or watching with the dead, is primarily an act of reverence and of sympathy. But "wakes," as we now mostly know them, are an abuse of this custom. […] Considerably over one hundred persons were present on one or other of the evenings on which these ceremonies were held, and, as the families were related, many attended the "Avakes" in both households. […] Of the persons present at the wakes here, four afterwards sickened. Among those attending the Thistle Street wake, six primary attacks resulted. The first illness in the Thistle Street household was pneumonic in type; and during the wakes three others of the family were sick, one of them of plague septicaemia. Seven families altogether were resident at 57 Thistle Street; but attacks occurred only among those who had been present at the wakes, although the importance of this may be to some extent discounted by the recognition of the nature of the disease five days after the death in this household occurred, and the consequent removal of all the known contacts to the reception-house[8].

With this observation, the authorities temporarily prohibited gatherings and visits during wakes.

As was usual for the Third Pandemic, rats were monitored during the outbreak. It was noted that:

> Rats were numerous in many of the infected tenements, and in those in which the type of the disease was pneumonic or intestinal, opportunities of infection, in all likelihood, occurred. On the recognition of the cases, inquiry failed to discover any evidence that rat-mortality prevailed to an unusual extent; and when a definite system of examination was begun, nearly three hundred, killed by trapping, or found dead in ashpits or elsewhere, chiefly within the area of infection, were bacteriologically examined without evidence of pest being discovered in any of them[8].

Interestingly, the authors of this report in 1901 already knew about the mechanisms of transmission mediated by ectoparasites: Fleas "together with flies, lice and ants, are capable of conveying the infection, and indirect contact may thus be established"[8].

While the sanitary commission made every attempt to understand the transmission of plague in Glasgow, the origins of the disease were still unclear. The commission wrote that, "The infection in the first outbreak in Glasgow in 1900 was no doubt imported into the city either by a human carrier of the disease or by infected material, more probably the latter, at a season of the year that was most favorable to the activity of the Bacillus pestis"[9]. They speculated on the origins that, "For this, modern methods of commerce and travel are responsible"[8] and, "Plague means so much to the mercantile and maritime interests of the town or city in which it may appear"[9].

Glasgow and Liverpool 1901. Although small in the number of cases, this outbreak is interesting because Colvin reconstructed the spread of plague between Glasgow and Liverpool, where eight cases occurred with six deaths. The outbreak began in August of 1901, in Glasgow, when it was reported that a 12-year-old boy became, "extremely ill, with a febrile temperature, and a painful swelling in his groin. No wounds or abrasion of any kind were seen on the boy's leg to account for the bubo. Two days later the boy's father took suddenly ill with the symptoms of an acute pneumonia […] He died suddenly after two days' illness. […] The house and his rag-store were disinfected and all the contacts removed to the sanitary reception house"[10]. Nevertheless, "at the end of October, 1901, there was a recrudescence of plague in Glasgow, four patients being found in the Central Station Hotel, while other two in association with them sickened of the same disease"[10]. These cases were connected to the 12-years old boy and his father, the ragman.

On August 15th 1901, a young woman in Glasgow developed a hidden mild form of plague with an iliac bubo, which was first diagnosed as an acute ovaritis[10]. During the week the woman was ill, two friends from Liverpool stayed three days with her. An account of the events stated that:

> Although they did not occupy the same bedroom, for there were five apartments in the house, they were in most intimate contact with the patient. On Sept 21st, or about four weeks later [when they were back to Liverpool], their mother sickened and died from plague after an illness of seven days with buboes in her axillae. On Sept. 22nd one of the girls sickened and died from plague nine days later with axillary buboes. On Sept. 24th the other girl sickened with plague with a bubo in her groin and she recovered. A woman who assisted in laying out the mother's body also died from plague, while four children living in an adjoining house sickened with plague, three of whom died[10].

Since the average incubation time of plague is about 10 days, Colvin could not at first establish a connection between the cases in Glasgow and those in Liverpool. Inquiries uncovered that, "the mother superintended the washing and laying aside of the clothes worn in Glasgow and thus caught the infection, and having evidently developed a virulent form of the disease infected her two daughters"[10]. The death of the four children in the neighboring house could be explained by the same mean of transmission:

> the very week that two of these children sickened their mother was wearing a blouse that had been given to her by one of the girls who had been to Glasgow, for the girl's mother being dead and the blouse being of a bright colour she could not wear it herself, for she was in mourning. The last time this blouse was worn by the girl was in Glasgow when in immediate contact with her friend, who was ill presumably with plague, for the blouse was never worn by her after she sickened with plague on account of her mother's death. I made strict inquiries whether the blouse had been washed or cleaned before being worn by the mother of the children and received a negative reply, for the blouse was silk and a new one and only worn in Glasgow[10].

Colvin reported other accounts of clothing being a carrier of plague: "many of the cases of plague in China were traced to the practice of the Chinese wearing the cloths of those who had died from the disease"[10]. He conveyed another interesting observation about immunity or asymptomatic cases of plague: a mother who spent 18 days with her daughter, a plague patient, slept with her and ate food handled by her without sickening[10].

Glasgow and Liverpool 1907. The third outbreak in the Scottish port occurred in 1907, again in August. "There were 2 known cases in the plague-infected area of 1900. In my opinion there were more cases, but I do not wish to introduce into this letter any disputed cases"[9]. The account of the outbreak was particularly interesting because it indicated that infected rats were involved:

> For the first time in any of the three outbreaks, infected rats were detected, and the disquieting fact was that they were found in Kinning Park, which is on the same side of the [river] Clyde, but fully a mile from the plague-infected area of 1900. These rats were accidentally discovered by giving rise to an offensive smell. They numbered 51, and had probably died about the same time. Only one of them was fit for bacteriological examination, and Dr. R.M. Buchanan, the city bacteriologist, reported (Local Government Board Report for Scotland, 1907) that the Bacillus pestis was found. A subculture proved virulent for a healthy mouse and a healthy rat within forty-eight and seventy-two hours respectively. Dr. Buchanan adds: 'In view of the absence of any other probable cause of the death of these rats it must be presumed that the others had all succumbed to plague' […] Finally, we have the second outbreak of plague in Liverpool – and again in autumn. […] Hence I would suggest the following relationship between each of the five outbreaks. There is not the shadow of a doubt that the other two outbreaks resulted from the first one and were not fresh importations into the city. They were a positive proof that the Bacillus pestis, as in all modern outbreaks, had remained in the city since 1900 in spite of all that was done to destroy it. The infection in the first outbreak of plague in Liverpool in 1901 was most probably brought into

> that city from Glasgow, as I have already described, by infected clothing, and in the absence of proof to the contrary I would now suggest that the infection in the recent three cases of plague in Liverpool is not a fresh importation, but is related in some way with the outbreak in Liverpool in 1901[9].

East Suffolk 1906-1918: John and Dorothy Black[11] reviewed the work of van Zwanenberg[12] on the progress of the small outbreaks that occurred in East Suffolk during 1906-1918. The first victim was a 9-year-old girl, who became ill with pneumonic plague in a cottage located five miles from Ipswich on the 13th of September 1910. Her mother died three days after her death, followed by her stepfather and a neighbor who had nursed her mother. It was written that, "All the victims had similar symptoms. The last two patients were buried on 30 September, the vicar taking the whole service in the open air; all those attending had their clothes disinfected. There were no necropsies or inquests. On 1 October the contacts were removed to isolation accommodation in Tattingstone Workhouse, which had been opened for this purpose."[11]. Some rats, a ferret, and a cat had also died close to the main river and their death was attributed to plague. A rat-survey was carried out in November 1910 and at the end of the year; the findings stated that:

> The investigators examined 568 captured rats; all were brown rats. Seventeen of these rats were found to be infected. […] Dr. Rowland paid particular attention to the flea population and obtained 584 fleas, about half of which were of the species *Nosopsyllus fasciatus*, which they demonstrated will readily bite man in the absence of its normal host. The stomachs of three fleas from rats infected with plague were examined; two contained a considerable number of plague bacilli. 40 rabbits were also examined, 2 of which carried the flea described above; 2 rabbits were found to be infected, one either recovering or suffering from chronic plague and one with acute plague[11].

A second, more extensive survey was carried out in January 1911, but the investigators did not find any infected rats. A third survey was organized between July and October 1911 and they found that, "Of 15 332 rats examined by dissection, 35 were found to be infected; diagnosis was mainly on the basis of post-mortem appearance and was confirmed by bacteriological culture in some cases. […] The surveys had shown that rats on both sides of the Orwell were infected"[11]. On October 10th, 1911, a sailor, based at the Royal Naval Barracks on the HMS Ganges in Shotley, developed severe pneumonia and an investigation of his sputum supported the diagnosis of plague. "He had cut himself while cleaning a rabbit which he had caught on the Ipswich Road […]. He recovered and died at the age of 76, although remained almost completely blind"[11].
Later rat campaigns, from 1912-1914, revealed that plague was sporadic:

> During 1912 a quarter of a million rats were killed but no cases of plague were discovered. In 1913 two parishes in the Shotley peninsula and one in the Woodbridge district were found to have infected rats, and 7 infected ferrets were found in the Woodbridge district. In 1914 no infected rats were found and no further action was taken because of the war[11].

Further inquiries retrospectively disclosed eight probable cases of pneumonic plague in 1906-1907, which had originally been certified as pneumonia:

> Dr Bulstrode was informed by a gamekeeper at Woolverstone Park [on the west bank of the river Orwell] that in 1906±1907 rats were observed to be dying in large numbers on the estate. The gamekeeper at Freston House reported a similar high mortality among rats in the autumn of 1910[11].

Another outbreak of bubonic plague was reported between December 1909 and January 1910:

> The infected family consisted of two adults and their five children, aged from 6 to 18 years. The home circumstances were poor and the house was reported to be infested with fleas. All seven members of the family were affected, of whom three recovered. All the victims developed bubonic plague, at intervals of three to six days between cases […]. Dr Bulstrode concluded that the family had suffered from bubonic plague, with case to case infection, probably by the human flea[11].

The last episodes of plague in East Suffolk concerned two women. The first became ill on Saturday June 8th, 1918[12] and died the following Thursday. Her neighbor who visited her died shortly after of pneumonic plague[12]. Their contacts were quarantined and all of their clothing and bedding was burned.

Due to the long-lasting presence of plague in the area, it was proposed that a reservoir was established in East Suffolk. It was written that, "There is no evidence that plague was in existence in Suffolk before 1906, nor were there any reports, apart from isolated cases in ports, of plague in other parts of the British Isles between 1906 and 1918"[11]. However, another explanation for plague in the area was that larger grain vessels coming from infected regions "off-loaded cargo into barges at Butterman's Bay on the north bank of the Orwell, to lighten their draught sufficiently to enable them to dock in Ipswich. It would have been easy for infected rats to swim ashore or for them to be brought on shore in sacks of grain"[11].
The number of rats coming off of ships was likely less after July 9th, 2012, when an ordinance in the United States introduced the use of rat guards for plague control. It was written that, "A rat guard is a sort of round metal "shield," placed over mooring lines to make it nearly impossible for rats to climb over and get onto or off the vessel when docked. Black rats were very common on all commercial ships from far back in history (and up to 1940s)"[13].

Catania 1914. In the newspaper *La Sicilia*, a short review appeared in 2014[14] about the report "La peste in Catania nel 1914". The report was written in 1917 by S. Privitera, a health official of Catania who helped to stop plague there in 1914. As stated in the report, plague was introduced by the steamer *Polcevera*, returning from Lybia. Infected rats were found on board and Privitera organized an extensive anti-rat campaign in the city. Eleven persons died in this outbreak of bubonic plague, including dockyard workers and their relatives. Among them was the daughter of a longshoreman, who had washed the clothes of her father.

Marseille 1900-1930. The work of Mafart et al.[15] is a valuable, rare account on the plague outbreaks in Marseille during the Third Pandemic:

> In 1900, 6 cases (no death) and 1901, 31 cases (4 deaths) were reported aboard ships coming from China, Egypt, Italy but the town was trusting their quarantine framework. So the first re-emergency of plague inside Marseille, in 1903 was a great surprise and cause for anxiety to local council and even, to national health authorities. […] At the end of August 1903, several deaths occurred among the workmen of a cardboard factory in city suburbs, at Saint-Barnabé district, which sorted old papers from Syria. Previously, rats, usually very many numerous in the factory, had disappeared and many rat corpses had been incinerated by the workmen. September 3th, a doctor noted the presence of bubo among two patients. The analysis of the pus imposed the diagnosis of plague. Most of patients were factory workers or parents of them. Suspects and subjects contacts (27 people) were hospitalized at the Salvator Hospital on September 6th with a rigorous bulk heading (Pons, 1904). An anti-plague serum was injected to the patients and the anti-plague vaccine was injected to 300 people, contacts and paramedical and medical personnel. The use of special garments (overall of fabric and Wellingtons) was imposed to paramedical and medical personnel. A sterilization with the drying oven of clothing was carried out. In spite of these precautions, three cases occurred among the personnel of the hospital. On the whole, 9 people died among 21 patients, 18

> contaminated downtowns, three at the Salvator hospital that was opened from September 6th to October 15th. This epidemic of plague in Marseilles was held secret and the national medical authorities sent the general inspector of Health to take the direction of prophylactic measurements. A disinfection of the buildings, houses of the patients and suspects was undertaken. The cardboard factory burned during the disinfection, which fire was recognized as voluntary in 1921. […] In 1913, a new case of plague was declared in the rebuilt factory. […] From 1919 to 1929, 132 cases of human plague were declared and involved 41 deaths […] There were 21 cases of plague (7 deaths) diagnosed among the sailors of the ships arriving or being at anchor […]. The employees working on the quays were exposed as well as the various trade associations, which approached the cargo warehouses (10 patients, 4 deaths). However, the majority of the cases of plague were described downtown among patients not having any relation with the port (101 cases, 30 deaths). These patients lived the unhealthiest districts of the city, at a few hundred miles from the port (Villette and Arenc district). In this part of town, where houses like a shantytown had no hygiene, occupied by poorest people, the rats were abounding […]. The epidemics generally began in a house or a slum. A person died with hot fever and some days later, others family members and neighbors were also ill and died. […] The captured or dead rats found in the port were sometimes infected. The presence of *Xenopsylla cheopis* was found among 92,7% among the rats captured on the ships, 33% among those of the quays and 50,4% among the rats captured downtown on a total of more than 9000 chips [*i.e.* fleas] examined in 1908 and 1909. Greatest epizootic was observed downtown in 1930: 28 among 42 infected rats discovered during the year in Marseilles among total amount 7275 examined rats came from the same district[15].

The authors concluded, "It is clearly proved that *Yersinia pestis* was present in urban murine population, contaminated for a long time by infected rodents living on harbor. So, at several time, in city areas were poverty allowed rodent increase, some sporadic bubonic plague human cases could occur with a secondary small outbreak, intensified by lack of hygiene and human fleas"[15].

Paris 1920-1. Although the French capital was already hit in 1917, there was a new outbreak of plague in Paris in 1920 – known as the "plague of the ragmen" (*peste des chiffonniers*). The outbreak was named after the majority of its victims who were ragmen living in conditions of extreme poverty, and it passed relatively unnoticed because of the earlier Spanish flu episode and the aftermath of the First World War[16]. This plague outbreak killed 33 people, with 95 reported cases[17]. The first known cases were children playing on the banks of the Seine, where suspicious barges were lodged[16].

Dublin 1921. We learn from Sir Arthur Ball[18] that he "was called to Sir Patrick Dun's Hospital late in the evening, to a case brought in by the ambulance, supposed to be one of strangulated hernia". He observed symptoms of a serious infection in the patient and decided to surgically remove a "gland" and send the specimen for analysis. Bubonic plague was diagnosed.

> Its mode of spreading may be by direct infection from one human being to another, either by inoculation with some discharge of the sick through a breach of surface in the healthy, or by inhalation of germ-laden atmosphere. By inoculation through the medium of rat fleas--which have left a sick rat and sought temporary sustenance from a human being. Sometimes the inoculation is caused by the bite of a sick rat or other animal. The case under consideration was that of a young woman of about 25 years, who […] lived not far from the shipping quays on the South side of the river, in a single room, alone, with a cat as bed-companion. When I saw her first on the morning of the 18th, I was at once struck with her typhus-like aspect. […] The trunk was covered with the marks of flea-bites, and the nurse informed me that she was in a very dirty state on admission. Careful search was made for the, minute, vesicle, or pustule, frequently seen at the site of inoculation on the macule made by a flea-bite, but nothing of the sort was found, and there was no wound to be found on the body[18].

She recovered after 13 days.

Barcelona 1931. After about 200 years' absence from the Iberian peninsula, plague struck on several occasions in Barcelona during the Third Pandemic: in 1905, with 52 cases and ten deaths; in 1919 with at least seven cases; in 1920 with a unique case; in October and November 1922, with a total of 28 cases; in November and December 1923, with two cases; in 1925, with one case ("in March, a man who brought a cargo of plantains from the Canary Islands"[19]); in October 1930, with four cases and four deaths; and in August-December 1931, with 31 cases, eight of which were fatal. The source of the infection, whether rats, goods or humans, could not be determined[19]. The most heavily hit quarters were the poorest, with unsanitary dwellings and refuse dumps in the vicinity. All measures to contain the outbreak were taken and rats were monitored as well during the outbreaks: 8,074 were examined, of which 4,268 bacteriologically (July 1931-January 1934). Only one rat was apparently infected, but inoculation tests using guinea pigs gave negative results. Of the total rats examined, over 99% were *R. norvegicus*, whereas of their 4,992 caught fleas, 1,985 were *X. cheopis* and 1,643 *C. fasciatus*[19].

Malta 1917. Malta's government, like many other European governments, feared the reintroduction of plague to the island, after the terrible outbreak of 1813[20] at the end of the Second Pandemic. Thus, in 1899, when plague was reported in Egypt and Portugal, the Maltese authorities authorized the Superintendent of Police to pay for every dead rat delivered. Over the course of one year, from November 1899 to November 1900, more than 49,400 rats were killed and delivered to the police[21]. Plague occurred again in Malta in 1917 and the first plague victim was, "infected from a sick rat which he found in a box containing stores coming from Mesopotamia where the disease was epidemic"[22]. With only eight cases and four deaths, the outbreak remained confined to the area around the port and occurred among dockyard workers and their contacts[22] from March 2nd to April 2nd, 1917[23]. "Of these cases, 7 were bubonic in form; 1 case was septicemic. Five of the 8 cases notified occurred at Calcara among a group of laborers from the neighboring island of Gozo, living in two tenements; the remaining cases occurred in contacts with this group"[23]. Over three months, Maj W. Broughton Alcock RAMC and Prof. Themistocles Zammit examined over 1,500 rats from around the Grand Harbor; of these, 15 rats were found to be infected[24]. The brown rat, *R. norvegicus,* was the predominant species in the neighborhood of the Grand Harbor. Their account stated that, "Other species were *M. rattus* (black rat), of more recent introduction and found also on the shore, and the variety *M. rattus alexandrinus*, which is fairly common in the island"[24]. The 102 fleas associated with the *R. rattus* individuals examined were: *X. cheopis* (60), *Ctenopsytta musculi* (38), *N. fasciatus* (3), and *Ctenocephalus* (1); whereas the 180 fleas taken from *R. decumanus* consisted of *X. cheopis* (118), *Ctenopsytta musculi* (49), *N. fasciatus* (3), and *Ctenocephalus* (10)[24]. Mites were also found on the rats, the most common being *Laelaps echidninus*[24].

Malta 1936-1937. Twenty years after the outbreak in 1917, a further epidemic occurred in Malta at Oormi from April 1936 to May 1937, which spread to Zebbug, with some additional cases in Rabat, Marsa, and Attard. In total, there were 33 cases and 12 deaths[25]. During 1936, the Health Department initiated an anti-rat campaign in the harbor areas, which led to the collection of 750 rats by trapping[21]. Plague was thought to have been imported by rodents that infested the hay and straw from the Barbary Coast[25]. Investigators found that an epizootic among *R. norvegicus* was present before the start of the epidemic[22]. *Leptopsylla segnis* was the most frequently found rat flea (48.75%), followed by *X. cheopis* (37.5%), whereas *N. fasciatus* was less common (13.75%)[22].

Malta 1945-1946. A further outbreak occurred in Malta from 1945-1946, in the commercial port area, which resulted in 80 cases and 22 deaths[26,27]. An account of the outbreak noted the involvement of rats and pets:

> From June 1945 to June 1946, out of 22,902 examined […] 20 rats were diagnosed as infected and of these 15 were *R. norvegicus*. It will be noticed that this species is clearly implicated as

> an important vector of plague in this outbreak […]. Although there was evidence of a widespread epizootic there was evidently a low incidence of infection; there were no reports of heavy mortality among rats which could be attributed to plague. Plague was also identified in one family of pet cavies and suspected in another. Both of the households concerned had human cases of plague as well[26].

Barnett further reported about the rat surveys carried out in those years:

> The obvious inference is that 4 months' intensive rat destruction had checked the plague outbreak. Unfortunately, it must be admitted that this inference is not safe one, since plague outbreaks always come to an end even if nothing is done to kill rats or their fleas. It cannot be proved that in this instance it was rat destruction that was responsible. However, the fact that the only cases of plague in the summer of 1946 were in an untreated village is suggestive[26].

Barnett described the conditions that led to the spillover of plague:

> As is usual in such outbreaks […], the majority of infected persons were accustomed to walking about in bare feet in filthy surrounding which provided harbourage for fleas. […] In Tower Road, Bubaqra, in which most of the Bubaqra cases lived, there were three privately owned refuse heaps. At one of these three *R. norvegicus* infested with bacteria indistinguishable from *P. pestis* were taken. Of 13 cases in Bubaqra, 3 were refuse collectors, and a fourth was a son of one of the 3; 5 others were associated in work, or topographically, with refuse collection[26].

The Maltese authorities also employed vector control against plague and, "From 1946 on, frequent and abundant use of DDT was introduced in Malta against insect and parasites, in particular against sand-fly which can transmit leishmaniosis and mosquitos. From 1948, the Insect Control section included a team of two labourers and one supervisor for the period April-November"[21].

Ajaccio 1945. The plague outbreak in Ajaccio occurred soon after World War II (May-July 1945), after centuries of absence from the island. The number of cases was limitied[28], but the death toll among the cases was relatively high. The Bull WHO 1951[22] wrote that plague was "apparently imported from North Africa". Additional information comes from a paper published in 1948[28], which said that the outbreak was confined to three small areas, one of which was a military barracks. Control measures were carried out, including compulsory vaccination for all the 25,000 citizens of Ajaccio. Of the 148 rats trapped after the outbreak, none were found to be infected. Of the rats that were trapped, 14 were *Rattus rattus alexandrinus* and the rest were *R. norvegicus*. They collected 101 fleas from the rats; 42 were *Xenopsylla cheopis*, of which all but eight were found on the 14 *R. rattus* individuals.

Taranto 1927 & 1945. During the Second Pandemic, the city of Taranto was struck by plague, in 1485 and again in 1523[29]. In the period of the Third Pandemic, a first lethal case of plague on a military vessel was reported in 1927 and did not produce any further victims[30,31]. At the beginning of September 1945, some dead mice were found in the harbor's armoury[32]. The first confirmed human plague case was reported on the 6th of September, and the last case was reported on the 29th of November[33]. All of the earliest victims were workers of the parcel office in the armoury[32], and the other cases lived in close proximity[32]. At that time, the official total number of cases was 29, of which 28 were civilian cases and one was among army personnel. With 15 deaths, the mortality rate was 51.7%; all 14 cases with primary septicemia died and one case out of 15 with primary bubonic plague died. No cases of pneumonic plague were reported. Seven of the cases that were reported had been inoculated; of these, three died of septicemic plague, the others with bubonic plague recovered[33]. Schultz[33] suggests that the exact source of infection was not clear:

> The disease may have existed in the form of a dormant epizootic in the Italian naval arsenal dock area for some considerable time before manifesting itself by human infection. Strong suspicion centred on a cargo of imported rags, stored in a shed in the arsenal, from which the infected rodents may have spread to other parts of the arsenal. The first cases notified had all been working in the vicinity of the shed, but, subsequently, infections occurred in persons situated in two other places. One of these persons was a military policeman on duty outside the arsenal, at a place where a broken drain might have given direct access to rodents, and the other was a civilian; it was not possible to trace the source of infection of the latter. The barque "Cherso" came under suspicion because the cargo of rags, which was stored in the shed and was later considered to be the primary source of infection, had been unloaded from it on about 28 July. The origin of the cargo is unknown; the ship may have come from Malta or some other port in an area where plague is endemic. […] Seizure of the ship was carried out when she arrived in Venice harbor on about 8 September. The results of the investigation are not known[33].

Schultz attested to receiving information on the movement of the ship from the Report ADMS 52 Army Area (obtained from UNRRA Health Division, Rome). More recent articles[32,34] summarizing the results of many years of historical research came to different conclusions about the origin of plague in Taranto. They claimed that plague was spread by an English mercantile transporting cotton noil from Malta. During the journey, the ship may have had an onboard fatal case of plague, which was not reported to the Italian authorities. Days before the first notification, the military police was seen at night quickly unloading a coffin onto one of their cars[32]. There were no official reports about the incident, but the British Army unofficially admitted that they had one case of plague[32]. The official number of victims of the epidemic is now considered to be 30.

This episode of plague occurred in Taranto after the end of World War II, when the Italian ports were partially still under the control of the allied military, as well as the civilian public-health organization[33]. The allied forces had imposed a veto on the dissemination of news about the plague outbreak[32,34]. Despite the difficult situation, the outbreak was stopped by officers of the Italian marines in only three months[32], with the help of the British military. A number of anti-plague measures were implemented; these included burning rags suspected of carrying plague, abundant spraying of DDT and notifications to the public[32]. It was written that, "The cases were immediately isolated, contacts were inoculated and kept under surveillance for 10 days, and their houses were sprayed with DDT and cleared of rodents"[33]. Perhaps due to the intervention measures, no relatives of the initial victims became ill. A large-scale anti-rodent campaign killed approximately 5,000 rats in three districts, with the help of two medical officers coming from India and two renowned specialists[32]. Of the rats that were poisoned in the docks, 60% were *R. norvegicus* and 40% were *R. rattus*. All the rats found in the city were black rats. None of the 308 rats tested for plague in 1946 were positive[33]. It is possible that only two rats tested in 1945 were positive for plague[34].

Reggio di Calabria 1946. At the beginning of January 1946, an isolated case of plague was reported in the port of Reggio di Calabria. Investigations showed that this was a case that was originally from Taranto[33].

References

1. Proust A. 1897 *La Défense de l'Europe contre la peste et la Conférence de Venise de 1897*. Masson, Paris, France.
2. Scheube B. 1908 *The diseases of warm countries: a handbook for medical men*. Bale & Danielsson, London, UK.
3. Echenberg M 2007 *Plague Ports: The Global Urban Impact of Bubonic Plague, 1894–1901*.

New York University Press, New York, USA.

4. Ferreira, M. E. C. 1981. Epidemias. *Dicionário História Port.* **2,** 406–408.

5. Kohn, G. C. 2007 *Encyclopedia of plague and pestilence: from ancient times to the present*. Third ediction. Facts on file, New York, USA.

6. Ribeiro, J. M. 1994 The City of Oporto at the End of the Nineteenth Century as Viewed by American Diplomats in Portugal. *Mediterr. Stud.* 143–156.

7. Irwin, F. 1899 Portugal. Plague conditions at Oporto—Continuation of letter published in Public Health Reports September 29. *Public Heal. Reports (1896-1970)* **14,** 2211–2213.

8. Chalmers AK. 1901 *Report on certain cases of plague occurring in Glasgow in 1900*. Robert Anderson. Glasgow, UK.

9. Colvin, T. 1908 Recent outbreaks of plague in Liverpool and Glasgow. *Br. Med. J.* **2,** 1782.

10. Colvin, T. 1907 Is Bubonic Plague still lurking in the City of Glasgow? *Lancet* **170,** 1522–1523.

11. Black, J. & Black, D. 2000 Plague in east Suffolk 1906-1918. *J. R. Soc. Med.* **93,** 540–543.

12. Van Zwanenberg, D. 1970 The last epidemic of plague in England? Suffolk 1906-1918. *Med. Hist.* **14,** 63–74.

13. Bramanti, B., Stenseth, N. C., Walløe, L. & Lei, X. 2016 Plague: A disease which changed the path of human civilization. in *Advances in Experimental Medicine and Biology* **918,** 1–26.

14. Giordano, G. 2014 Il funzionario onesto e sconosciuto che nel 1914 fermò la peste a Catania. *La Sicilia* 19.

15. Mafart B, Louis FJ, Matton T. 2007 Plague in Marseille within 20th century. In: Signoli M, editor. *Plague: epidemics and societies* p. 31–34 Firenze University Press, Florence, Italy.

16. Barry S, Gualde N. 2010 *La troisième pandémie de peste et les épidémies contemporaines: une nouvelle menace planétaire. Démographie et santé, Actes du XIVe colloque national de démographie Bordeaux - 21 au 24 mai 2007*, CUDEP, Bordeaux, France, 363 – 374.

17. Signoli, M. 2017 Reflections on crisis burials related to past plague epidemics. *Clin. Microbiol. Infect.* **18,** 218–223.

18. Ball, A. & Drury, H. C. 1921 A case of bubonic plague in Dublin. *Dublin J. Med. Sci.* **2,** 63–67.

19. Castella PC, Collado JG. 1934 *Estudio de las ratas y de sus ectoparásitos en ocasión del brote epidémico de peste en Barcelona en 1931*. Commision Permanente de Investigaciones Sanitarias, Madrid, Spain.

20. Calvert, R. 1815 An Account of the origin and progress of the Plague of Malta, in the year 1813. *Med. Chir. Trans.* **6,** 1.

21. Savona-Ventura, C. 2016 *Contemporary Medicine in Malta [1798-1979]*. Lulu.com.

22. Pollitzer, R. 1951 Plague studies: I. A summary of the history and a survey of the present distribution of the disease. *Bull. World Health Organ.* **4,** 475.

23. 1922 Prevalence of Disease: Foreign and Insular. *Public Heal. Reports* **37,** 75–82.

24. Zammit T. 1918 Rats and parasites in plague epidemics. *Arch. Melitensis* **3**, 141-143.

25. Malta Garrison 1945. Medical Officers of the Malta Garrison. Available at: http://maltaramc.com/regsurg/rs1940_1949/rmo1945.html.

26. Barnett, S. A. 1948 Rat control in a plague outbreak in Malta. *Epidemiol. Infect.* **46,** 10–18.

27. Berger, S. 2017 *Infectious Diseases of Malta 2017 edition*. GIDEON Informatics Inc, Los Angeles, USA.
28. Bernard, L. & Dounet, G. 1948 An Epidemic of Bubonic Plague at Ajaccio (1945). *Recl. Trav. l'Institut Natl. d'Hygiene* **2,** 355–375.
29. Vacca, N. 1959 La peste a Taranto nel 1523 e il trattato di Epifanio Ferdinando sulla malattia. *La Zagaglia Rass. di Sci. Lett. ed arti* **A.I,** 12–21.
30. Leccisotti, G. 1957 La peste bubbonica a Taranto nell'autunno 1945. *Rass. e Boll. di Stat. Comune di Taranto* **26,** 15.
31. Berger, S. 2017 *Infectious Diseases of the Canary Islands 2017 edition*. GIDEON Informatics Inc, Los Angeles, USA.
32. Leone, A. 2000. Taranto fra guerra e dopoguerra. Il minamento della Rada di Mar Grande (1943) e l'episodio epidemico di peste bubbonica (1945). *Cenacolo* **12 n.s. 2,** 149–188.
33. Schulz, K. H. 1950 Control of plague in Taranto, Italy, 1945/1946: An account of a successful programme of rodent extermination. *Bull. World Health Organ.* **2,** 673.
34. Carducci A. 2001 L'ultima peste in Europa: Taranto 1945. In Atti del XLI Congresso Nazionale della Societa` Italiana di Storia della Medicina (ed. AE Distante, ML Portulano-Scoditti), pp. 163 – 176. Giordano Editore, Mesagne (Brindisi), Italy.
